# Supplementary material for: Conditional chemoconnectomics (cCCTomics) as a strategy for efficient and conditional targeting of chemical transmission
Source: eLife. 2024 Apr 30;12:RP91927. doi: 10.7554/eLife.91927 (PMC11060718; doi:10.7554/eLife.91927)
Supplement: Figure 3—source data 3. [file elife-91927-fig3-data3.zip › Figure 3 source data3 part 1.zip!/Figure 3-Source data 3-part1/1063-13-CRISPResso_Report_8cz_fsdr.zip!/CRISPResso_on_1063_14/1c.Alignment_barplot.pdf]

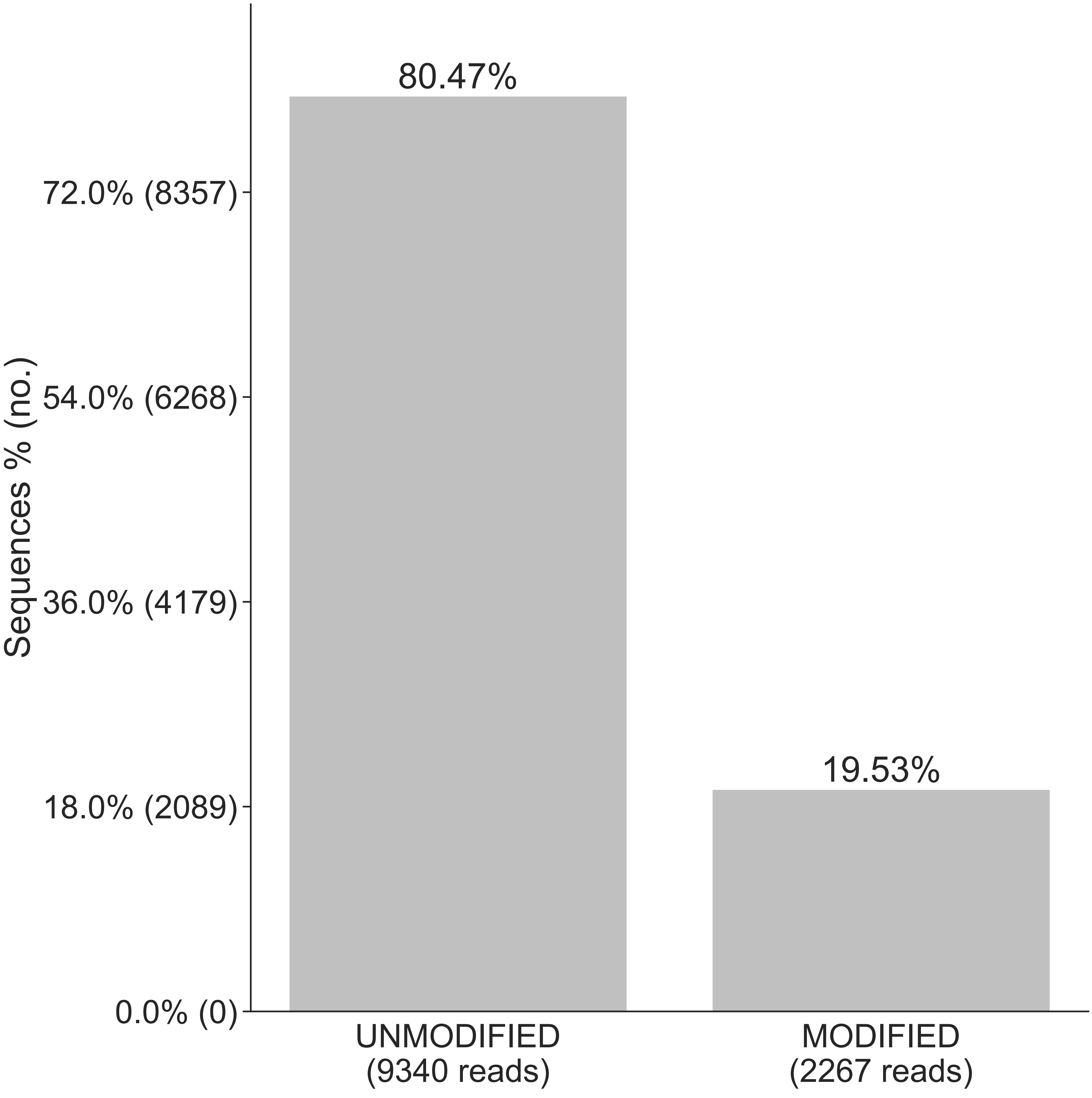
80.47%
19.53%
72.0% (8357)
54.0% (6268)
36.0% (4179)
18.0% (2089)
0.0% (0)
Sequences % (no.)
UNMODIFIED
(9340 reads)
MODIFIED
(2267 reads)
